# Chapter 10: Mining Genome-Wide Genetic Markers

**DOI:** 10.1371/journal.pcbi.1002828

**Published:** 2012-12-27

**Authors:** Xiang Zhang, Shunping Huang, Zhaojun Zhang, Wei Wang

**Affiliations:** 1 Department of Electrical Engineering and Computer Science, Case Western Reserve University, Ohio, United States of America; 2 Department of Computer Science, University of North Carolina at Chapel Hill, North Carolina, United States of America; 3 Department of Computer Science, University of California at Los Angeles, California, United States of America; Whitehead Institute, United States of America; University of Maryland, Baltimore County, United States of America

## Abstract

Genome-wide association study (GWAS) aims to discover genetic factors underlying phenotypic traits. The large number of genetic factors poses both computational and statistical challenges. Various computational approaches have been developed for large scale GWAS. In this chapter, we will discuss several widely used computational approaches in GWAS. The following topics will be covered: (1) An introduction to the background of GWAS. (2) The existing computational approaches that are widely used in GWAS. This will cover single-locus, epistasis detection, and machine learning methods that have been recently developed in biology, statistic, and computer science communities. This part will be the main focus of this chapter. (3) The limitations of current approaches and future directions.

**What to Learn in This Chapter**

- The background of Genome-wide association study (GWAS).
- The existing computational approaches that are widely used in GWAS. This will cover single-locus, epistasis detection, and machine learning methods.
- The limitations of current approaches and future directions.

This article is part of the “Translational Bioinformatics” collection for *PLOS Computational Biology*.

## 1. Introduction

With the advancement of genotyping technology, genome-wide high-density single nucleotide polymorphisms (SNPs) of human and other organisms are now available [1], [2]. The goal of genome-wide association studies (GWAS) is to seek strong associations between phenotype and genetic variations in a population that represent (genomically proximal) causal genetic effects. As the most abundant source of genetic variation, millions of SNPs have been genotyped across the entire genome. Analyzing such large amount of markers poses great challenges to traditional computational and statistical methods. In this chapter, we introduce the basic concept of genome-wide association study, and discuss recently developed methods for GWAS.

Genome-wide association study is an inter-discipline problem of biology, statistics and computer science [3], [4], [5], [6]. In this section, we will first provide a brief introduction to the necessary biological background. We will then formalize the problem and discuss both traditional and recently developed methods for genome-wide analysis of associations.

A human genome contains over 3 billion DNA base pairs. There are four possible nucleotides at each base in the DNA: adenine (A), guanine (G), thymine (T), and cytosine (C). In some locations in the genome, a genetic variation may be found which involves two or more nucleotides across different individuals. These genetic variations are known as *single-nucleotide polymorphism* (SNPs), i.e., a variation of a single nucleotide in the DNA sequence. In most cases, there are two possible nucleotides for a variant. We denote the more frequent one as “0”, and the less frequent one as “1”. For bases on autosomal chromosomes, there are two parallel nucleotides, which leads to three possible combinations, “00”, “01” and “11”. These genotype combinations are known as “major homozygous site”, “heterozygous site” and “minor heterozygous site” respectively. These genetic variations contribute to the phenotypic differences among the individuals. (A phenotype is the composite of an organism's observable characteristics or traits.) Genome-wide association study (GWAS) aims to find strong associations between SNPs and phenotypes across a set of individuals.

More formally, let 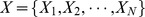 be the set of 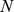 SNPs for 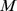 individuals in the study, and 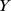 be the phenotype of interest. The goal of GWAS is to find SNPs (markers) in 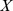, that are highly associated with 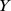. There are several challenging issues that need to be addressed when developing an analytic method for GWAS [7], [8].

**Scalability** Most GWAS datasets consist of a large number of SNPs. Therefore the algorithms for GWAS need to be highly scalable. For example, for a typical human GWAS, the dataset may contain up to millions SNPs and involve thousands of individuals. Inefficient methods may consume a large amount of computational resources and time to find highly associated SNPs.

**Missing markers** Even with the current dense genotyping technique, many genetic variants are still not genotyped. Current methods usually assume genetic linkage to enhance the power. Imputation, which tries to impute the unknown markers by using existing SNPs databases, is another popular approach to handle missing markers. The well known related projects include the International HapMap project [9] and the 1000 Genomes Project [10].

**Complex traits** One approach in GWAS is to test the association between the trait and each marker in a genome, which is successful in detecting a single gene related disease. However, this approach may have problems in finding markers associated with complex traits. This is because that complex traits are affected by multiple genes, and each gene may only have a weak association with the phenotype. Such markers with low marginal effects are hard to detect by the single-locus methods.

In the remainder of the chapter, we will first discuss the single-locus methods. We will then study epistasis detection (multi-locus) approaches which are designed for association studies of complex traits. For epistasis detection, we will mainly focus on exact two-locus association mapping methods.

## 2. Single-Locus Association Mapping

As the rapid development of high-throughput genotyping technology, millions of SNPs are now available for genome-wide association studies. Single-locus association test is a traditional way for association studies. Specifically, for each SNP, a statistical test is performed to evaluate the association between the SNP and the phenotype. A variety of tests can be applied depending on the data types. The phenotype involved in a study can be case-control (binary), quantitative (continuous), or categorical. We categorize the statistical tests based on what kind of phenotypes they can be applied on.

### 2.1 Problem Formalization

Let 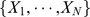 be a set of 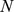 SNPs for 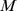 individuals and 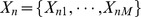 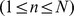. We use 0, 1, 2 to represent the homozygous major allele, heterozygous allele, and homozygous minor allele respectively. Thus we have that 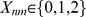 (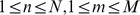). Let 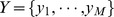 be the phenotype. Note that the values that 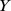 can take depend on its type.

### 2.2 Case-Control Phenotype

In a case-control study, the phenotype can be represented as a binary variable with 0 representing controls and 1 representing cases.

A contingency table records the frequencies of different events. Table 1 is an example contingency table. For a SNP 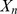 and a phenotype 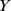, and we use 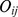 to denote the number of individuals whose 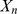 equals 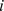 and 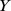 equals 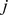. Also, we have 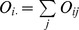 and 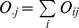 . The total number of individuals 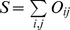 .

**Table 1:** Contingency table for a single SNP 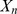 and a phenotype 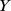.

	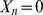	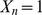	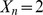	Totals
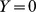	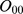	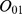	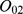	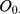
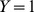	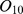	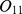	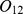	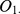
**Totals**	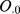	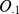	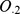	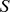

Many tests can be used to assess the significance of the association between a single SNP and a binary phenotype. The test statistics are usually based on the contingency table. The null hypothesis is that there is no association between the rows and columns of the contingency table.

#### 2.2.1 Pearson's 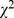 test

Pearson's 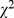 test can be used to test a null hypothesis stating that the frequency distribution of certain events observed in a sample is consistent with a particular theoretical distribution [11].

The value of the test statistic is
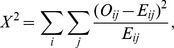
where 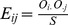. The degree of freedom is 2.

#### 2.2.2 G-test

G-test is an approximation of the log-likelihood ratio. The test statistic is
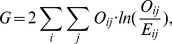
where 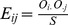.

The null hypothesis is that the observed frequencies result from random sampling from a distribution with the given expected frequencies. The distribution of G is approximately that of 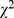, with the same degree of freedom as in the corresponding 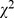 test. When applied to a reasonable size of samples, the G-test and the 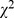 test will lead to the same conclusions.

#### 2.2.3 Fisher exact test

When the sample size is small, the Fisher exact test is useful to determine the significance of the association. The p-value of the test is the probability of the contingency table given the fixed margins. The probability of obtaining such values in Table 1 is given by the hypergeometric distribution:
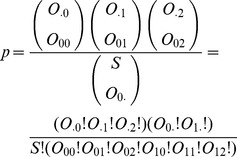
Most modern statistical packages can calculate the significance of Fisher tests. The actual computation performed by the existing software packages may be different from the exact formulation given above because of the numerical difficulties. A simple, somewhat better computational approach relies on a gamma function or log-gamma function. How to accurately compute hypergeometric and binomial probabilities remains an active research area.

#### 2.2.4 Cochran-Armitage test

For complex traits, contributions to disease risk from SNPs are widely considered to be roughly additive. In other words, the heterozygous alleles will have an intermediate risk between two homozygous alleles. Cochran-Armitage test can be used in this case [12], [5]. Let the test statistic of U be the following:
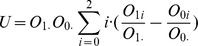
After substitution, we get
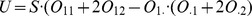
The variance of U under the null hypothesis can be computed as
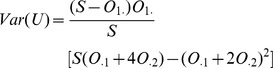
Notice that for a large sample size 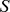, we have 
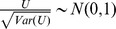
, hence 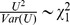.

#### 2.2.5 Summary

There is no overall winner of the introduced tests. Cochran-Armitage test may not be the best if the risks are deviated from the additive model. Meanwhile, 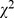 test, G-test, and Fisher exact test can handle the full range of risks, but they will unavoidably lose some power in the detection of additive ones. Different tests may be applied on the same data to detect different effects.

### 2.3 Quantitative Phenotype

In addition to case-control phenotypes, many complex traits are quantitative. This type of study is also often referred to as the quantitative trait locus (QTL) analysis. The standard tools for testing the association between a single marker and a continuous outcome are analysis of variance (ANOVA) and linear regression.

#### 2.3.1 One-way ANOVA

The F-test in one-way analysis of variance is used to assess whether the expected values of a quantitative variable within several pre-defined groups differ from each other.

For each SNP 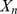, we can divide all the individuals into three groups according to their genotypes. Let 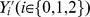 be a subset of phenotypes of which the individuals have the genotypes equal to 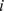. We represent the number of phenotypes in 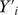 as 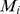, and we have 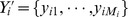. Notice that 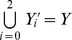 and 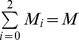


The total sum of squares (SST) can be divided into two parts, the between-group sum of squares (SSB) and the within-group sum of squares (SSW):
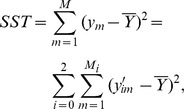


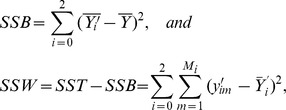
where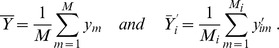 The formula of F-test statistic is 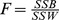, and F follows the F-distribution with 2 and S-3 degrees of freedom under the null hypothesis, i.e., 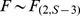.

#### 2.3.2 Linear regression

In the linear regression model, a least-squares regression line is fit between the phenotype values and the genotype values [11]. For simplicity, we denote the genotypes of a single SNP to be 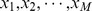. Based on the data 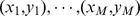, we need to fit a line in the form of 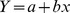.

We have the sums of squares as follows:
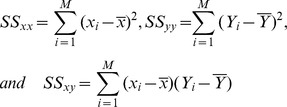
where 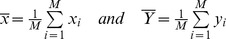


To achieve least squares, the estimator of 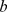 is 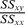. To evaluate the significance of the obtained model, a hypothesis testing for 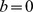 is then applied.

### 2.4 Multiple Testing Problem

In a typical GWAS, the test needs to be performed many times. We should pay attention to a statistical issue known as the multiple testing problem. In the remainder of this section, we will discuss the multiple testing problem and how to effectively control error rate in GWAS.

Type 1 error rate, is the possibility that a null hypothesis is rejected when it is actually true. In other words, it is the chance of observing a positive (significant) result even if it is not. If a test is performed multiple times, the overall Type 1 Error rate will increase. This is called the multiple testing problem.

Let 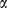 be the type 1 error rate for a statistical test. If the test is performed 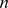 times, the experimental-wise error rate 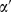 is given by
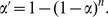
For example, if 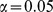 and 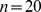, then 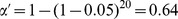. In this case, the chance of getting at least one false positive is 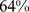.

Because of the multiple testing problem, the test result may not be that significant even if its p-value is less than a significant level 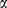. To solve this problem, the nominal p-value need to be corrected/adjusted.

### 2.5 Family-Wise Error Rate Control

For the single-locus test, we denote the p-value for a association test of a SNP 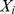 and a phenotype 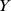 to be 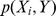, and the corrected p-value to be 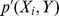. Family-wise error rate (FWER), or the experiment-wise error rate, is the probability of at least one false association. We use 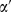 to denote family-wise error rate, and it is given by
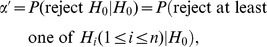
where 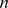 is the total number of tests and 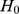 is the hypothesis that all the 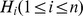 are true.

Many methods can be used to control FWER. Bonferroni correction is a commonly used method, in which p-values need to be enlarged to account for the number of comparisons being performed. Permutation test [13] is also widely used to correct for multiple testing in GWAS.

#### 2.5.1 Bonferroni correction

In Bonferroni correction, the p-value of a test is multiplied by the number of tests in the multiple comparison.
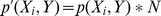
Here the number of tests is the number of SNPs 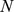 in a study. Bonferroni correction is a single-step procedure, in which each of the p-values is independently corrected.

#### 2.5.2 Permutation tests

In the permutation test, data are reshuffled. For each permutation, p-values for all the tests are re-calculated, and the minimal p-value is retained. After 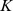 permutations, we get totally 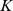 minimal p-values. The corrected p-value is given by the proportion of minimal p-values which is less than the original p-value.

Let 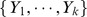 be the set of 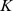 permutations. For each permutation 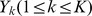, the minimal p-value 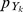 is given by
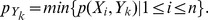
Then we have the corrected p-value
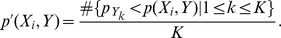
The permutation method takes advantage of the correlation structure between SNPs. It is less stringent than Bonferroni correction.

### 2.6 False Discovery Rate Control

False discovery rate (FDR) controls the expected proportion of type 1 error among all significant hypotheses. It is less conservative than the family-wise error rate. For example, if 100 observed results are claimed to be significant, and the FDR is 0.1, then 10 of results are expected to be false discoveries.

One way to control the FDR is as follows [14]. The p-values of SNPs and the phenotype are ranked from smallest to largest. We denote the ordered p-values to be 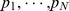. Starting from the largest p-value to the smallest, the original p-value is multiplied by the total number of SNPs and divided by its rank. For the 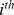 p-value 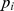, its corrected p-value 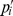 is given by
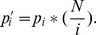
In this section, we have discussed commonly used methods in single-locus study, the multiple testing problem and how to control error rate in GWAS. In the next section, we will introduce methods used for two-locus association studies. We will focus on one class work that finds exact solution when searching for SNP-SNP interactions in GWAS.

## 3. Exact Methods for Two-Locus Association Study

The vast number of SNPs has posed great computational challenge to genome-wide association study. In order to understand the underlying biological mechanisms of complex phenotype, one needs to consider the joint effect of multiple SNPs simultaneously. Although the idea of studying the association between phenotype and multiple SNPs is straightforward, the implementation is nontrivial. For a study with total 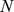 SNPs, in order to find the association between 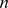 SNPs and the phenotype, a brute-force approach is to exhaustively enumerate all 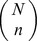 possible SNP combinations and evaluate their associations with the phenotype. The computational burden imposed by this enormous search space often makes the complete genome-wide association study intractable. Moreover, although permutation test has been considered the gold standard method for multiple testing correction, it will dramatically increase the computational burden because the process needs to be performed for all permuted data.

In this section, we will focus on the recently developed exact method for two-locus epistasis detection. Different from the single-locus approach, the goal of two-locus epistasis detection is to identify interacting SNP-pairs that have strong association with the phenotype. FastANOVA [15] is an algorithm for two-locus ANOVA (analysis of variance) test on quantitative traits and FastChi [16] for two-locus chi-square test on case-control phenotypes. COE [17] is a general method that can be applied in a wide range of tests. TEAM [18] is designed for studies involving a large number of individuals such as human studies. In this subsection, we will discuss these algorithms, and their strengths and limitations.

### 3.1 The FastANOVA Algorithm

FastANOVA utilizes an upper bound of the two-locus ANOVA test to prune the search space. The upper bound is expressed as the sum of two terms. The first term is based on the single-SNP ANOVA test. The second term is based on the genotype of the SNP-pair and is independent of permutations. This property allows to index SNP-pairs in a 2D array based on the genotype relationship between SNPs. Since the number of entries in the 2D array is bound by the number of individuals in the study, many SNP-pairs share a common entry. Moreover, it can be shown that all SNP-pairs indexed by the same entry have exactly the same upper bound. Therefore, we can compute the upper bound for a group of SNP-pairs together. Another important property is that the indexing structure only needs to be built once and can be reused for all permutated data. Utilizing the upper bound and the indexing structure, FastANOVA only needs to perform the ANOVA test on a small number of candidate SNP-pairs without the risk of missing any significant pair. We discuss the algorithm in further detail in the following.

Let 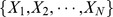 be the set of SNPs of 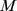 individuals (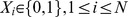) and 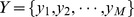 be the quantitative phenotype of interest, where 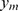 (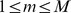) is the phenotype value of individual 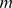.

For any SNP 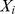 (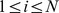), we represent the F-statistic from the ANOVA test of 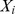 and 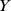 as 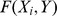. For any SNP-pair 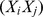, we represent the F-statistic from the ANOVA test of 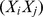 and 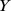 as 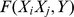.

The basic idea of ANOVA test is to partition the total sum of squared deviations 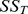 into between-group sum of squared deviations 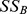 and within-group sum of squared deviations 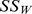:
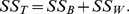
In our application of the two-locus association study, Table 2 and Table 3 show the possible groupings of phenotype values by the genotypes of 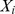 and 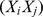 respectively.

**Table 2:** Grouping of 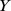 by 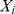.

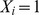	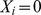
group 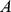	group 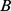

**Table 3:** Grouping of 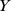 by 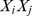.

	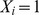	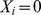
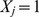	group 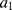	group 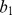
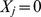	group 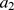	group 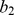

Let 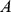, 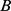, 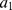, 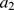, 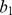, 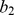 represent the groups as indicated in Table 2 and Table 3. We use 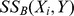 and 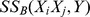 to distinct the one locus (i.e., single-SNP) and two locus (i.e., SNP-pair) analyses. Specifically, we have
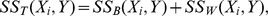


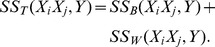
The F-statistics for ANOVA tests on 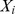 and 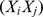 are:
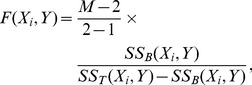
(1.1)

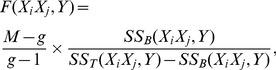
(1.2)where 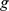 in Equation (1.2) is the number of groups that the genotype of 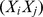 partitions the individuals into. Possible values of 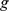 are 3 or 4, assuming all SNPs are distinct: If none of groups 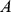, 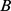, 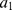, 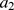, 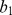, 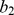 is empty, then 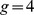. If one of them is empty, then 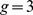.

Let 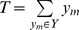 be the sum of all phenotype values. The total sum of squared deviations does not depend on the groupings of individuals:
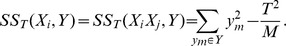
Let 
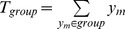
 be the sum of phenotype values in a specific group, and 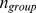 be the number of individuals in that group. 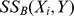 and 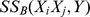 can be calculated as follows:
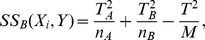


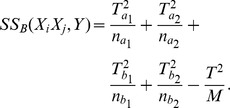
Note that for any group of 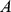, 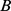, 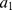, 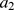, 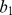, 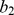, if 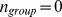, then 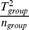 is defined to be 0.

Let 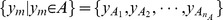 be the phenotype values in group 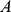. Without loss of generality, assume that these phenotype values are arranged in ascending order, i.e.,
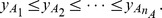
Let 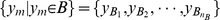 be the phenotype values in group 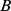. Without loss of generality, assume that these phenotype values are arranged in ascending order, i.e.,
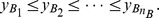
We have the overall upper bound on 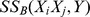:

**Theorem 1** *(Upper bound of* 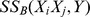 *)*

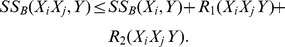



The notations in the bound can be found in Table 4. The upper bound in Theorem 1 is tight. The tightness of the bound is obvious from the derivation of the upper bound, since there exists some genotype of SNP-pair 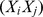 that makes the equality hold.

**Table 4:** Notations for the bounds.

Symbols	Formulas
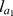	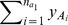
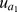	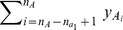
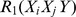	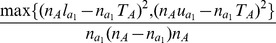
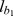	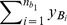
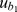	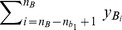
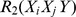	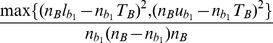

We now discuss how to apply the upper bound in Theorem 1 in detail. The set of all SNP-pairs is partitioned into non-overlapping groups such that the upper bound can be readily applied to each group. For every 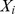 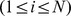, let 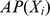 be the set of SNP-pairs
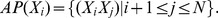
For all SNP-pairs in 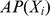, 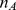, 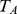, 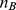, 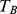 and 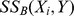 are constants. Moreover, 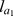, 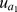 are determined by 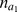, and 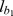, 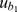 are determined by 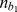. Therefore, in the upper bound, 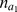 and 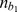 are the only variables that depend on 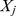 and may vary for different SNP-pairs 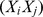 in 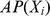.

Note that 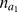 is the number of 1's in 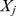 when 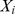 takes value 1, and 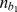 is the number of 1's in 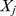 when 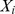 takes value 0. It is easy to prove that switching 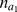 and 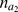 does not change the F-statistic value and the correctness of the upper bound. This is also true if we switch 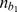 and 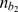. Therefore, without loss of generality, we can always assume that 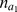 is the smaller one between the number of 1's and number of 0's in 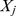 when 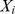 takes value 1, and 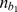 is the smaller one between the number of 1's and number of 0's in 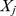 when 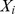 takes value 0.

If there are 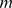 1's and 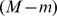 0's in 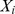, then for any 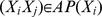, the possible values that 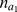 can take are 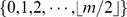. The possible values that 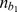 can take are 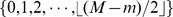.

To efficiently retrieve the candidates, the SNP-pairs 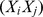 in 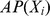 are grouped by their 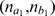 values and indexed in a 2D array, referred to as 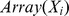.

**Figure 1:**
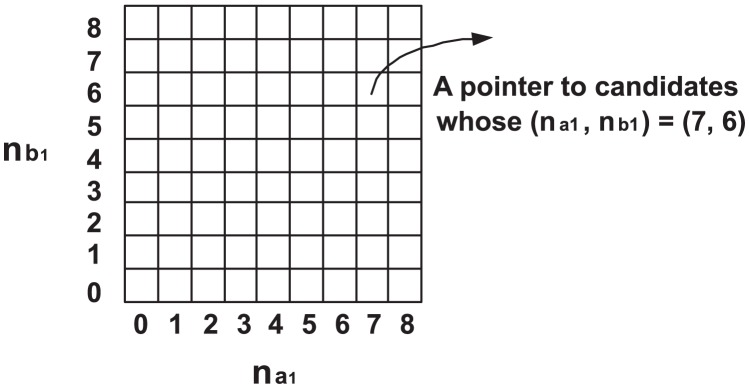
The index array 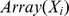 for efficient retrieval of the candidate SNP-pairs.

Suppose that there are 32 individuals, and the genotype of 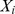 consists of half 0's and half 1's. Thus for the SNP-pairs in 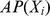, the possible values of 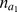 and 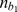 are 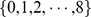. Figure 1 shows the 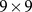 array, 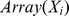, whose entries represent the possible values of 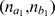 for the SNP-pairs 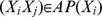. The entries in the same column have the same 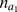 value. The entries in the same row have the same 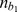 value. The 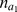 value of each column is noted beneath each column. The 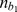 value of each row is noted left to each row. Each entry of the array is a pointer to the SNP-pairs 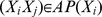 having the corresponding 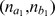 values.

For any SNP 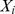, the maximum number of the entries in 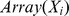 is 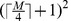. The proof of this property is straightforward and omitted here. In order to find candidate SNP-pairs, we scan all entries in 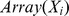 to calculate their upper bounds. Since the SNP-pairs indexed by the same entry share the same 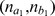 value, they have the same upper bound. In this way, we can calculate the upper bound for a group of SNP-pairs together. Note that for typical genome-wide association studies, the number of individuals 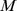 is much smaller than the number of SNPs 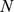. Therefore, the additional cost for accessing 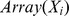 is minimal compared to performing ANOVA tests for all pairs 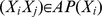.

For multiple tests, permutation procedure is often used in genetic analysis for controlling family-wise error rate. For genome-wide association study, permutation is less commonly used because it often entails prohibitively long computation times. Our FastANOVA algorithm makes permutation procedure feasible in genome-wide association study.

Let 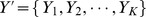 be the 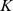 permutations of the phenotype 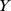. Following the idea discussed above, the upper bound in Theorem 1 can be easily incorporated in the algorithm to handle the permutations. For every SNP 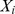, the indexing structure 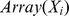 is independent of the permuted phenotypes in 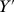. The correctness of this property relies on the fact that, for any 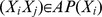, 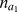 and 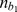 only depend on the genotype of the SNP-pair and thus remain constant for different phenotype permutations. Therefore, for each 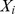, once we build 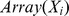, it can be reused in all permutations.

### 3.2 The FastChi Algorithm

As our initial attempt to develop scalable algorithms for genome-wide association study, FastANOVA is specifically designed for the ANOVA test on quantitative phenotypes. Another category of phenotypes is generated in case-control study, where the phenotypes are binary variables representing disease/non-disease individuals. Chi-square test is one of the most commonly used statistics in binary phenotype association study. We can extend the principles in FastANOVA for efficient two-locus chi-square test. The general idea of FastChi is similar to that of FastANOVA, i.e., re-formulating the chi-square test statistic to establish an upper bound of two-locus chi-square test, and indexing the SNP-pairs according to their genotypes in order to effectively prune the search space and reuse redundant computations. Here we briefly introduce the FastChi algorithm.

For SNP 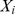, we represent the chi-square test value of 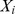 and the binary phenotype 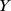 as 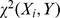. For any SNP-pair 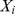 and 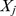, we use 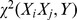 to represent the chi-square test value for the combined effect of 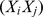 with 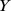. Let 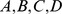 represent the following events respectively: 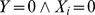; 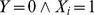; 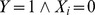; 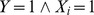. Let 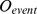 denote the observed value of an event. 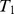, 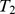, 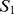, 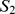, 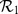, and 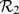 represent the formulas shown in Table 5. We have the upper bound of 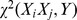 stated in Theorem 2.

**Table 5:** Notations used in the derivation of the upper bound for two-locus Chi-square test.

Symbols	Formulas
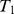	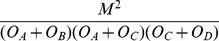
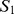	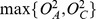
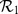	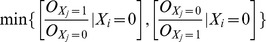
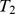	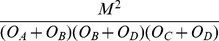
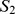	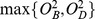
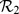	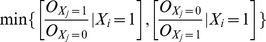

**Theorem 2** *(Upper bound of* 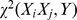 *)*

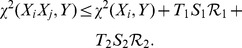



For given phenotype 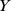 and SNP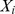, 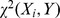, 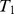, 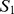, 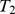, and 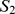 are constants. 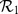 and 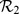 are the only variables that depend on 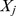 and may vary for different SNP-pairs 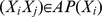. (Recall that 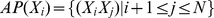.) Thus for a given 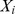, we can treat equation 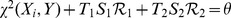 as a *straight line* in the 2-D space of 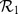 and 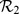. The ones whose 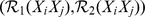 values fall below the line can be pruned without any further test.

**Figure 2:**
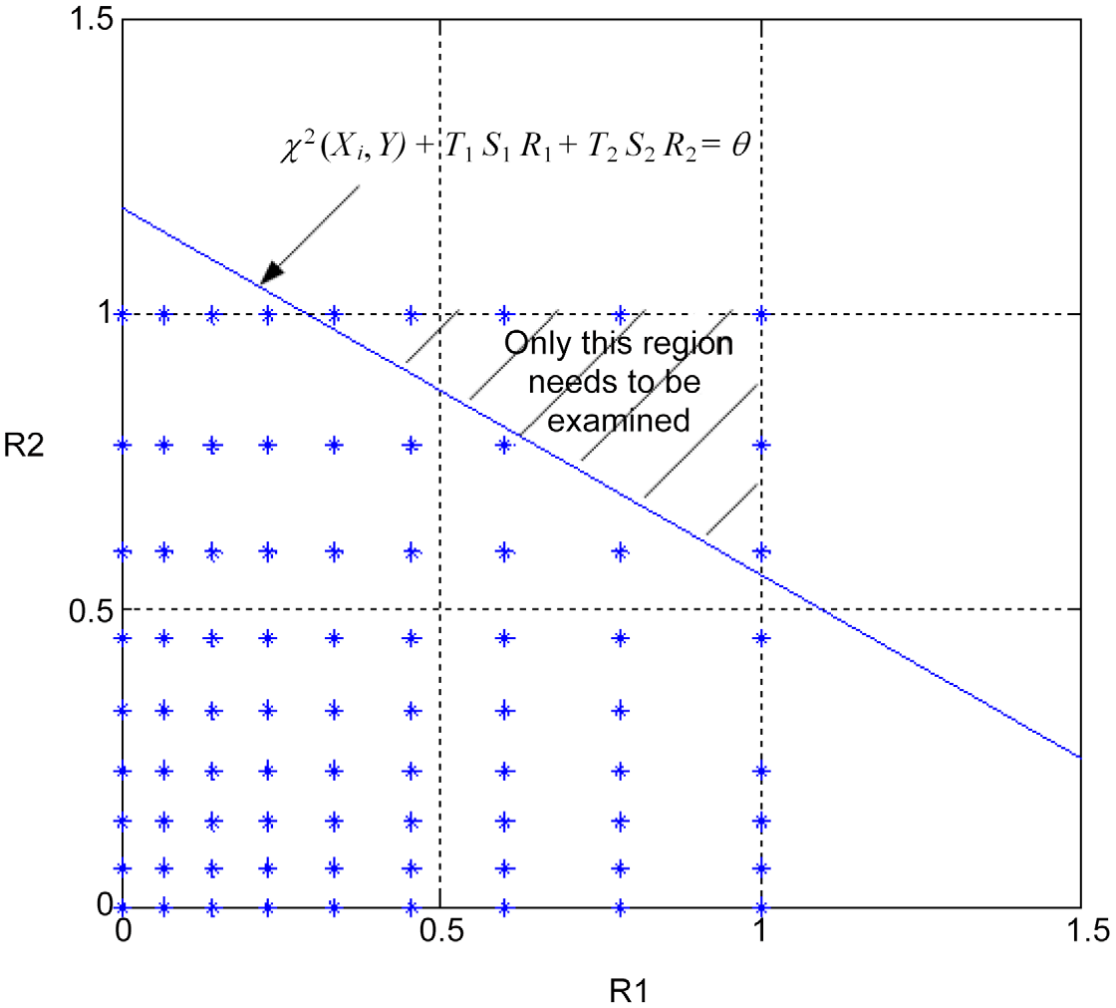
Pruning SNP-pairs in 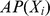 using the upper bound.

Suppose that there are 32 individuals, 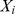 contains half 0's, and half 1's. For the SNP-pairs in 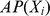, the possible values of 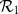 (and 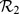) are 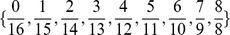. Figure 2 shows the 2-D space of 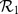 and 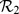. The blue stars represent the values that 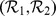 can take. The line 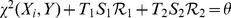 is plotted in the figure. Only the SNP-pairs whose 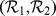 values are in the shaded region are subject to two-locus Chi-square test.

Similar to FastANOVA, in FastChi, we can index the SNP-pairs in 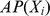 according to their genotype relationships, i.e., by the values of 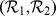. Experimental results demonstrate that FastChi is an order of magnitude faster than the brute force alternative.

### 3.3 The COE Algorithm

Both FastANOVA and FastChi rework the formula of ANOVA test and Chi-square test to estimate an upper bound of the test value for SNP pairs. These upper bounds are used to identify candidate SNP pairs that may have strong epistatic effect. Repetitive computation in a permutation test is also identified and performed once those results are stored for use by all permutations. These two strategies lead to substantial speedup, especially for large permutation test, without compromising the accuracy of the test. These approaches guarantee to find the optimal solutions. However, a common drawback of these methods is that they are designed for specific tests, i.e., chi-square test and ANOVA test. The upper bounds used in these methods do not work for other statistical tests, which are also routinely used by researchers. In addition, new statistics for epistasis detection are continually emerging in the literature. Therefore, it is desirable to develop a general model that supports a variety of statistical tests.

The COE algorithm takes the advantage of convex optimization. It can be shown that a wide range of statistical tests, such as chi-square test, likelihood ratio test (also known as G-test), and entropy-based tests are all convex functions of observed frequencies in contingency tables. Since the maximum value of a convex function is attained at the vertices of its convex domain, by constraining on the observed frequencies in the contingency tables, we can determine the domain of the convex function and get its maximum value. This maximum value is used as the upper bound on the test statistics to filter out insignificant SNP-pairs. COE is applicable to all tests that are convex.

### 3.4 The TEAM Algorithm

The methods we have discussed so far provide promising alternatives for GWAS. However, there are two major drawbacks that limit their applicability. First, they are designed for relatively small sample size and only consider homozygous markers (i.e., each SNP can be represented as a 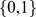 binary variable). In human study, the sample size is usually large and most SNPs contain heterozygous genotypes and are coded using 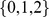. These make previous methods intractable. Second, although the family-wise error rate (FWER) and the false discovery rate (FDR) are both widely used for error controlling, previous methods are designed only to control the FWER. From a computational point of view, the difference in the FWER and the FDR controlling is that, to estimate FWER, for each permutation, only the maximum two-locus test value is needed. To estimate the FDR, on the other hand, for each permutation, all two-locus test values must be computed.

To address these limitations, TEAM is proposed for efficient epistasis detection in human GWAS. TEAM has several advantages over previous methods. It supports to both homozygous and heterozygous data. By exhaustively computing all two-locus test values in permutation test, it enables both FWER and FDR controlling. It is applicable to all statistics based on the contingency table. Previous methods are either designed for specific tests or require the test statistics satisfy certain property. Experimental results demonstrate that TEAM is more efficient than existing methods for large sample studies.

TEAM incorporates the permutation test for proper error controlling. The key idea is to incrementally update the contingency tables of two-locus tests. We show that only four of the eighteen observed frequencies in the contingency table need to be updated to compute the test value. In the algorithm, we build a minimum spanning tree [19] on the SNPs. The nodes of the tree are SNPs. Each edge represents the genotype difference between the two connected SNPs. This tree structure can be utilized to speed up the updating process for the contingency tables. A majority of the individuals are pruned and only a small portion are scanned to update the contingency tables. This is advantageous in human study, which usually involves thousands of individuals. Extensive experimental results demonstrate the efficiency of the TEAM algorithm.

As a summary of the exact two-locus algorithms, FastANOVA and FastChi are designed for specific tests and binary genotype data. The COE algorithm is a more general method that can be applied to all convex tests. The TEAM algorithm is more suitable for large sample human GWAS.

## 4. Multifactor Dimensionality Reduction

Multifactor dimensionality reduction (MDR) [20] is a data mining method to identify interactions among discrete variables for binary outcomes. It can be used to detect high-order gene-gene and gene-environment interactions in case-control studies. By pooling multi-locus SNPs into two groups, one classified as high-risk and the other classified as low risk, MDR effectively reduces the predictors from 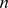 dimensions to one dimension. Then, the one-dimensional variable is evaluated through cross-validation. The steps are repeated for all other 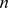 factor combinations, and the factor model which has the lowest prediction error is chosen as the ‘best’ 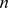 factor model. Its detailed steps are as follows:

- Divide the set of factors into 10 equal subsets.
- Select a set of 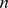 factors from the pool of all factors in the training set
- Create a contingency table for these 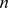 factors by counting the number of cases and controls in each combination.
- Compute the case-control ratio in each combination. Label them as “high-risk if it is greater than a certain threshold, and otherwise, it is marked as “low-risk”.
- Use the labels to classify individuals. Compute the misclassification rate.
- Repeat previous steps for all combinations of 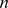 factors across 10 training and testing subsets.
- Choose the model whose average misclassification rate is minimized and cross-validation consistency is maximized as the “best” model.

MDR designs a constructive induction method that combines two or more SNPs before testing for association. The power of the MDR approach is that it can be combined with other methodologies including the ones described in this chapter.

## 5. Logistic Regression

Logistic regression is a statistical method for predicting binary and categorical outcome. It is widely used in GWAS [21], [22]. The basic idea is to use linear regression to model the probability of the occurrence of a specific outcome. Logistic regression is applicable to both single-locus and multi-locus association studies and can incorporate covariates and other factors in the model.

Let 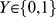 be a binary variable representing disease status (diseased verses non diseased), and 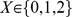 be a SNP. The conditional probability of having the disease given a SNP is 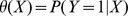. We define the logit function to convert the range of the probability from 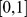 to 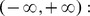


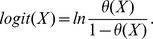
The logit can be considered as a latent continuous variable that will be fit to a linear predictor function:
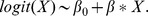
To cope with multiple SNP loci and potential covariates, we can modify the above model. For example, in the following model the logit is fit with predictors of SNPs (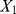, 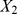) and covariates (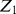, 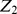):
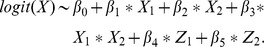
Although logistic regression can handle complicated models, it may be computationally demanding when the number of predictors is large [23].

## 6. Summary

The potential of genome-wide association study for the identification of genetic variants that underlying phenotypic variations is well recognized. The availability of large SNP data generated by high-throughput genotyping methods poses great computational and statistical challenges. In this chapter, we have discussed serval computational approaches to detect associations between genetic markers and the phenotypes. For further readings, the readers are encouraged to refer to [11], [7], [24], [25] for discussions about current progress and challenges in large-scale genetic association studies.

## 7. Exercises

**Question 1:** The table below contains binary genotype and case-control phenotype data from ten individuals. Give the contingency table and use 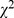 test to compute the association test score.
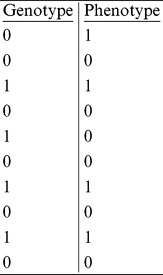



**Question 2:** Assuming that we have the following SNP and phenotype data, is the SNP significantly associated with the phenotype? Here, we represent each SNP site as the number of minor alleles on that locus, so 0 and 2 are for major and minor homozygous sites, respectively, and 1 is for the heterozygous sites. We also assume that minor alleles contribute to the phenotype and the effect is additive. In other words, the effect from a minor homozygous site should be twice as large as that from a heterozygous site. You may use any test methods introduced in the chapter. How about permutation tests?
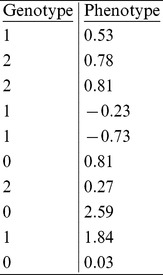



**Question 3:** Categorize the following methods in the table. The methods are 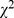 test, G-test, ANOVA, Student's T-test, Pearson's correlation, linear regression, logistic regression.
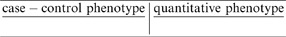



**Question 4:** Why is it important to study multiple-locus association? What are the challenges?

Answers to the Exercises can be found in Text S1.

**Further Reading**

- Cantor RM, Lange K, Sinsheimer JS (2008) Prioritizing GWAS results: a review of statistical methods and recommendations for their application. Nat Rev Genet 9(11): 855–867.
- Cordell HJ (2009) Detecting gene-gene interactions that underlie human diseases. Nat Rev Genet 10(6): 392–404.
- Manolio TA, Collins FS, Cox NJ, Goldstein DB, Hindorff LA, et al. (2009) Finding the missing heritability of complex diseases. Nature 461(7265): 747–753.
- Moore JH, Williams SM (2009) Epistasis and its implications for personal genetics. Am J Hum Genet 85(3): 309–320.
- Phillips PC (2010) Epistasis - the essential role of gene interactions in the structure and evolution of genetic systems. Am J Hum Genet 86(1): 6–22.
- Wang K, Li M, Hakonarson H (2010) Analysing biological pathways in genome-wide association studies. Nat Rev Genet 11: 843–854.

## Supporting Information

Text S1 Answers to Exercises
(PDF)
