# Supplementary material for: Chapter 10: Mining Genome-Wide Genetic Markers
Source: PLoS Comput Biol. 2012 Dec 27;8(12):e1002828. doi: 10.1371/journal.pcbi.1002828 (PMC3531292; doi:10.1371/journal.pcbi.1002828)
Supplement: Text S1 — Answers to Exercises (PDF) [file pcbi.1002828.s001.pdf]

Mining Genome-wide Genetic Markers

The Supporting Information legend: Text S1: Answers to Exercises

Xiang Zhang, Shunping Huang, Zhaojun Zhang and Wei Wang

Question 1: The table below contains binary genotype and case-control phenotype data from ten individuals. Give the contingency table and use χ^2 test to compute the association test score.

Genotype	Phenotype
0	1
0	0
1	1
0	0
1	0
0	0
1	1
0	0
1	1
0	0

Answer:

	Genotype = 0	Genotype = 1
Phenotype = 0	5	1
Phenotype = 1	1	3

The test statistics of χ^2 test is 1.41.

Question 2: Assuming that we have the following SNP and phenotype data, is the SNP significantly associated with the phenotype? Here, we represent each SNP site as the number of minor alleles on that locus, so 0 and 2 are for major and minor homozygous sites, respectvely, and 1 is for the heterozygous sites. We also assume that minor alleles contribute to the phenotype and the effect is additive. In other words, the effect from a minor homozygous site should be twice as large as that from a heterozygous site. You may use any test methods introduced in the chapter. How about permutation tests?

Genotype	Phenotype
1	0.53
2	0.78
2	0.81
1	-0.23
1	-0.73
0	0.81
2	0.27
0	2.59
1	1.84
0	0.03

Answer:

Take the linear regression with F-test as an example, the p-value without the permutation test is 0.54, and is 0.55 after 1000 permutation tests (this number could be different with your answer because of the randomness in the permutation test).

Question 3: Categorize the following methods in the table. The methods are χ^2 test, G-test, ANOVA, Student's T-test, Pearson's correlation, linear regression, logistic regression.

case-control phenotype	quantitative phenotype

Answer:

case-control phenotype	quantitative phenotype
χ^2 test, G-test	Student's T-test, ANOVA
logistic regression	linear regression
Pearson's correlation	Pearson's correlation

Question 4: Why is important to study multiple-locus association? What are the challenges?

Answer:

Many diseases are known to be complex traits. In order to reveal the true mechanism, it is better to study association between phenotype and multiple markers.

The multiple-locus association study poses both statistical challenges and computation challenges.
